# Cost-effectiveness analysis of omeprazole for preventing esophageal stricture in patients with Zargar grade 2b and 3a corrosive esophageal injuries: A trial-based economic evaluation

**DOI:** 10.1097/MD.0000000000050701

**Published:** 2026-09-11

**Authors:** Prasit Mahawongkajit, Saritphat Orrapin, Prakitpunthu Tomtitchong, Chittinad Havanond

**Affiliations:** a Department of Surgery, Faculty of Medicine, Thammasat University, Pathum Thani, Thailand.

**Keywords:** caustic injury, corrosive esophageal injury, cost-effectiveness analysis, esophageal stricture, omeprazole

## Abstract

**Background:**

Corrosive esophageal injury frequently results in esophageal stricture requiring repeated endoscopic dilatation and substantial healthcare expenditure. This study evaluated the cost-effectiveness of omeprazole plus standard treatment compared with standard treatment alone for preventing esophageal stricture in adult patients with Zargar grade 2b and 3a corrosive esophageal injuries.

**Methods:**

A trial-based economic evaluation was conducted alongside a randomized controlled trial from the healthcare provider and patient perspectives. Twenty patients were randomized to receive either standard treatment alone (n = 10) or standard treatment plus omeprazole (n = 10). Direct medical costs were analyzed using the incremental cost-effectiveness ratio. Deterministic one-way sensitivity analysis and probabilistic sensitivity analysis using Monte Carlo simulation were performed.

**Results:**

The incidence of corrosive esophageal stricture was 20% (2/10) in the omeprazole group and 70% (7/10) in the standard treatment group (relative risk, 0.29; 95% confidence interval, 0.08–1.05; Fisher’s exact test, *P* = .070). Omeprazole plus standard treatment reduced healthcare costs by THB 4642.30 per patient from the provider perspective and THB 5476.60 per patient from the patient perspective. The intervention remained the dominant strategy across all deterministic sensitivity analyses. Probabilistic sensitivity analysis demonstrated that 68.3% and 78.8% of simulations favored omeprazole from the provider and patient perspectives, respectively.

**Conclusion:**

Omeprazole plus standard treatment may represent a cost-effective strategy for adult patients with Zargar grade 2b and 3a corrosive esophageal injuries. However, these findings should be considered preliminary and require confirmation in larger multicenter randomized controlled trials.

## 1. Introduction

Injuries to the esophagus are particularly concerning, with perforation and stricture being major complications.^[1–6]^ Researchers are now working on the best practices for detecting transmural necrosis and perforation to avoid unnecessary surgery.^[3,7,8]^ After the acute phase, patients with esophageal injuries of Zargar grade 2b and 3a are at risk for developing strictures, which can cause suffering and reduce their quality of life.^[9–11]^ Therefore, the prevention and early diagnosis of strictures, as well as effective treatments, are important areas of study for the late sequelae.^[4,6,10,11]^

Preventing complications from strictures is crucial for patients with caustic injuries, as it impacts both their health and finances. Cost-effectiveness analysis is an economic evaluation method for the value of money used for health intervention compared to the clinical outcome gained. We previously demonstrated that omeprazole plus standard treatment could decrease the rate of esophageal stricture compared with the usual standard treatment alone.^[10]^ However, physicians or policymakers may be unconvinced that the intervention would be worth the implementation. Therefore, alongside that trial, we conducted an economic analysis. The main purpose of this study was to evaluate whether standard treatment plus omeprazole is cost-effective compared to standard treatment alone for the prevention of corrosive esophageal stricture, using data from our previous randomized controlled trial (RCT).

## 2. Materials and methods

### 2.1. Study design

This was a post hoc trial-based economic evaluation conducted from the healthcare provider and patient perspectives alongside a previously completed RCT with a four-week follow-up period. The economic evaluation was not prespecified in the original trial protocol. Complete details of the clinical trial have been reported previously.^[10]^

### 2.2. Setting

The study took place from April 2018 to January 2020 at Thammasat University hospital, Pathum Thani, Thailand.

### 2.3. Interventions

A study was conducted to compare the effectiveness of omeprazole plus standard treatment with standard treatment alone. For the omeprazole plus standard treatment group, patients were given 80 mg/day of omeprazole intravenously for 3 days followed by 40 mg/day orally for 4 weeks without administered corticosteroids or antibiotics. The routine care included nil per os with intravenous fluid and close clinical monitoring. Patients also received consultations with psychiatrists, psychologists, and social workers. Drinking water was allowed starting 24 to 48 hours after the endoscopic examination, while clear liquids were started depending on clinical symptoms. Oral intake was gradually increased to a full liquid or soft diet with sufficient calories once the patient’s condition improved. Before discharge, a multidisciplinary team prepared the patient and their family for a home program. Four weeks post-discharge, patients underwent a barium swallow esophagogram to check for an esophageal stricture. All films were reviewed by experienced diagnostic radiologists. Patients who had radiological evidence of a corrosive esophageal stricture underwent an esophagogastroduodenoscopy to confirm the diagnosis. The physician who assessed the clinical outcomes of the study was blinded to whether the patients received omeprazole or not. All patients with confirmed esophageal strictures underwent esophageal dilatation every 1 to 2 weeks for treatment by Savary-Gilliard dilator with Fluoroscopy or Balloon Dilatation.

### 2.4. Participants

Twenty adult patients (12 males and 8 females; mean age 29.4 ± 6.6 years) with Zargar grade 2b and 3a corrosive esophageal injuries were randomized to either standard care (n = 10) or standard care plus omeprazole (n = 10).

### 2.5. Ethics approval

This study was approved by the Human Research Ethics Committee, Faculty of Medicine, Thammasat University (Approval No. MTU-EC-SU-0-035/61). The study was conducted in accordance with the ethical principles of the Declaration of Helsinki. Written informed consent was obtained from all participants prior to enrollment.

### 2.6. Health outcome measure

Effectiveness was defined as the incidence of corrosive esophageal stricture in each treatment group.

### 2.7. Cost data

This study analyzed the cost of healthcare services from both the providers’ and patients’ perspectives. Only direct medical costs were considered for the analysis. The hospital’s cost evaluating center provided the unit costs to value the resources used from the providers’ perspective. The analysis included capital costs, material costs, and labor costs, as shown in Table 1. Clinical staff completed case record forms to track the use of theater resources and other resources during the patients’ admission and until they were discharged from the hospital. After discharge, follow-up physician visits were scheduled at the out-patient department.

**Table 1:** Major service items and direct medical costs.

Service items	Providers’ perspective (THB)	Patients’ perspective (THB)
**Hospitalization**		
Emergency department	321.91 per visit	120 per visit
Out-patient department	484.00 per visit	0
General ward	1428.00 per 24 h	400 per 24 h
Endoscopic room	862.76 per 1 h	120 per person
**Procedure**		
Upper GI study	1218.14 per person	1800 per person
EGD	2341.86 per person	2000 per person
Balloon Dilatation	1662.00 per person	8311 per person
Savary-Gilliard dilator with Fluoroscopy	3015.07 per person	1700 per person
**Drug**		
Omeprazole 40mg intravenous route	47.05 per vial	72.00 per vial
Omeprazole Cap 20 mg	1.12 per capsule	1.50 per capsule
10 % Triamcinolone 1 mL	11.39 per unit	64 per unit

1 USD = 31.2959 Baht exchange rate at January 31, 2020.

EGD = Esophagogastroduodenoscopy, THB = Thai Baht, USD = United States dollar.

### 2.8. Statistical analysis

The analysis was conducted using trial data from randomization until the end of the fourth week follow-up period. As the follow-up period was less than 1 year,^[12]^ cost discounting was not necessary. Cost-effectiveness was determined by identifying the different costs incurred for additional health outcomes due to the patient’s management.^[13]^ The cost-effectiveness results were presented by incremental cost-effectiveness ratio (ICER), which was calculated using the following formula:

ICER = (C1–C2)/(E1–E2).

C1: Cost of omeprazole plus standard treatment group

C2: Cost of standard treatment group

E1: Esophageal stricture rate in the omeprazole plus standard treatment group

E2: Esophageal stricture rate in the standard treatment group

Lower esophageal stricture rates were considered to indicate greater treatment effectiveness.

The economic analysis was performed using Microsoft Excel. Results were evaluated using deterministic and probabilistic sensitivity analyses.

### 2.9. Sensitivity analysis

All of the model’s parameters are uncertain and subject to variation. To determine the model’s robustness, a conventional one-way sensitivity analysis was conducted. In this analysis, each parameter’s value was tested by varying it within a plausible range. Due to the cost distribution’s skewness, the cost ranges were varied from 10% below to 3 times above the base case estimation. Additionally, we conducted a probabilistic sensitivity analysis. This analysis involved using the gamma distribution to generate random values for all unit costs and the beta distribution for health outcome parameters.^[14,15]^

## 3. Results

The study analyzed the cost-effectiveness of omeprazole plus standard treatment compared with standard treatment alone. The total expenditure per service item and per patient is presented in Table 1. Patients who received omeprazole plus standard treatment had lower mean costs and a lower incidence of corrosive esophageal stricture than those who received standard treatment alone. Specifically, the incidence of corrosive esophageal stricture was 20% (2/10) in the omeprazole plus standard treatment group and 70% (7/10) in the standard treatment group (relative risk, 0.29; 95% confidence interval, 0.08–1.05; Fisher’s exact test, *P* = .070), corresponding to the prevention of 5 strictures for every ten patients treated.

From the provider perspective, omeprazole plus standard treatment reduced healthcare costs by 4642.30 Baht per patient compared with standard treatment alone. From the patient perspective, the intervention resulted in cost savings of 5476.60 Baht per patient, as shown in Table 2.

**Table 2:** Base case results of health impact.

Intervention	Total cost (THB per case)	Total health outcome endpoint (corrosive esophageal stricture rate)	ICER*
Provider perspective			
Omeprazole plus standard care	12,103.32	0.20	
Standard care	16,745.62	0.70	Dominated**
Difference mean costs	−4642.30		
Patient perspective			
Omeprazole plus standard care	7652.60	0.20	
Standard care	13,129.20	0.70	Dominated**
Difference mean costs	−5476.6		

USD = 31.2959 Baht exchange rate at January 31, 2020.

ICER = incremental cost-effectiveness ratio; THB = Thai Baht; USD = United States dollar.

* Incremental cost-effectiveness ratio.

** Dominated strategy: Standard care was more costly and less effective than omeprazole plus standard care.

**Figure 1.**
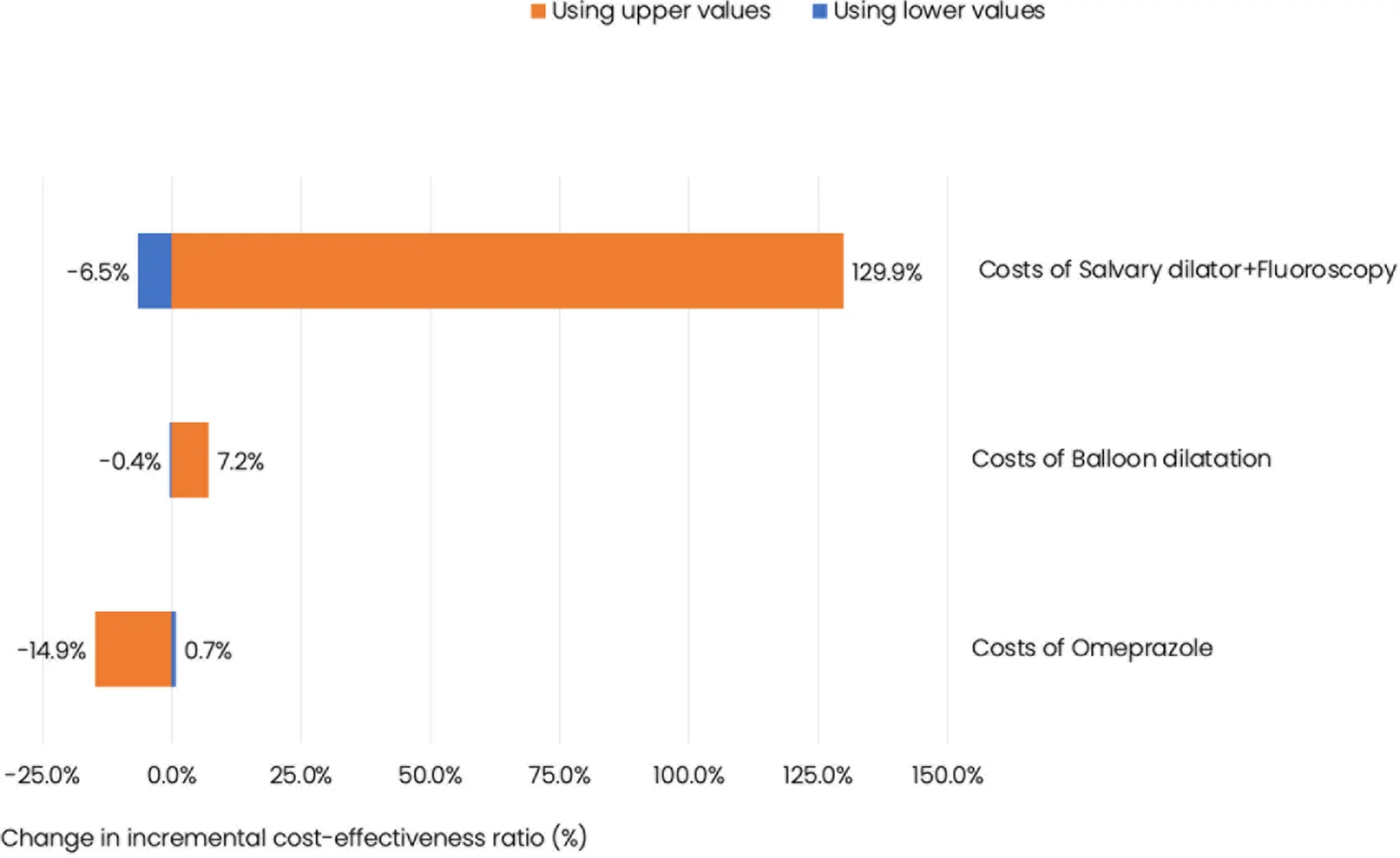
Tornado diagram. Tornado diagram illustrating the results of providers’ perspectives using one-way sensitivity analysis.

**Figure 2.**
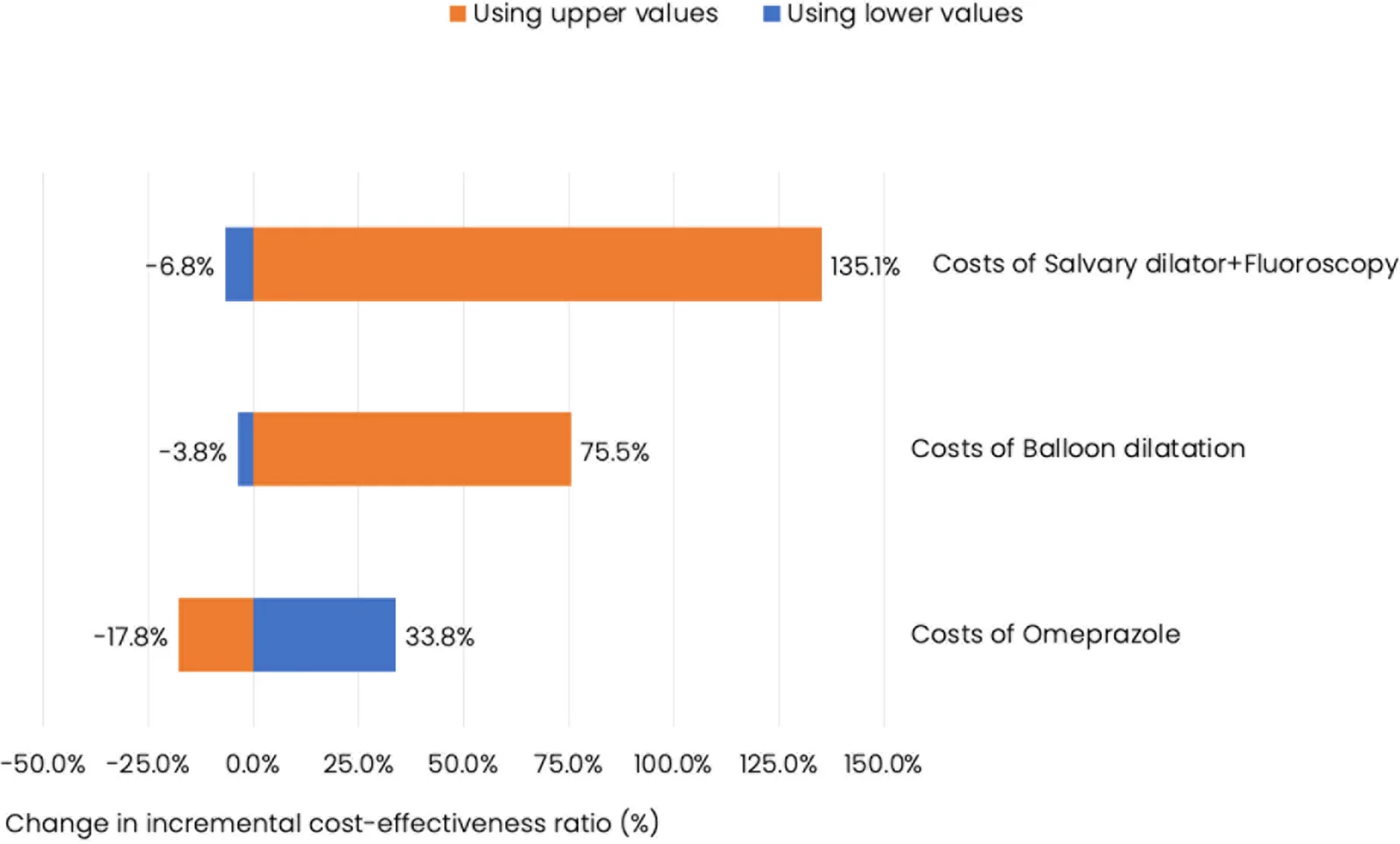
Tornado diagram illustrating the results of patients’ perspective using one-way sensitivity analysis.

Even when key medical costs were varied, omeprazole plus standard treatment remained the dominant strategy, as demonstrated by the one-way sensitivity analysis (Table 3) and the Tornado diagrams (Figs. 1 and 2). The ICER was most sensitive to the costs of balloon dilatation and Savary-Gilliard dilatation under fluoroscopic guidance.

**Table 3:** Results of one-way sensitivity analysis.

Providers’ perspective				
**Key parameters (Base case (THB**))	**Range for sensitivity analysis**	**Total cost in omeprazole plus standard treatment arm**	**Total cost in standard treatment arm**	**ICER** *
Balloon dilatation (1662.00)	1495.80–4986.00	12,086.71–12,435.73	16,712.39–17,410.43	−9251.36–−9949.40
Savary-Gilliard dilator + Fluoroscopy (3015.07)	2713.56–9045.21	12,043.03–13,309.36	16,383.82–23,981.80	−8681.58–−21,344.88
Omeprazole (345.02)	310.52–1035.06	12,068.83–12,793.37	16,745.62–16,745.62	−9353.58–−7904.50

Patients’ perspective				
Key parameters (Base case (THB))	Range for sensitivity analysis	Total cost in omeprazole plus standard treatment arm	Total cost in standard treatment arm	ICER*
Balloon dilatation (10,311.00)	9279.90–30,933.00	7445.74–11,789.80	12,715.48–21,403.60	−10,539.48–−19,227.60
Savary-Gilliard dilator + Fluoroscopy (3700.00)	3330.00–11,100.00	7578.60–9132.60	12,685.20–22,009.20	−10,213.20–−25,753.20
Omeprazole (516.00)	464.40–1548.00	5801.00–8684.60	13,129.20–13,129.20	−14,656.40–−8999.20

USD = 31.2959 Baht exchange rate at January 31, 2020.

ICER = incremental cost-effectiveness ratio, THB = Thai Baht, USD = United States dollar.

* Incremental cost-effectiveness ratio.

**Figure 3.**
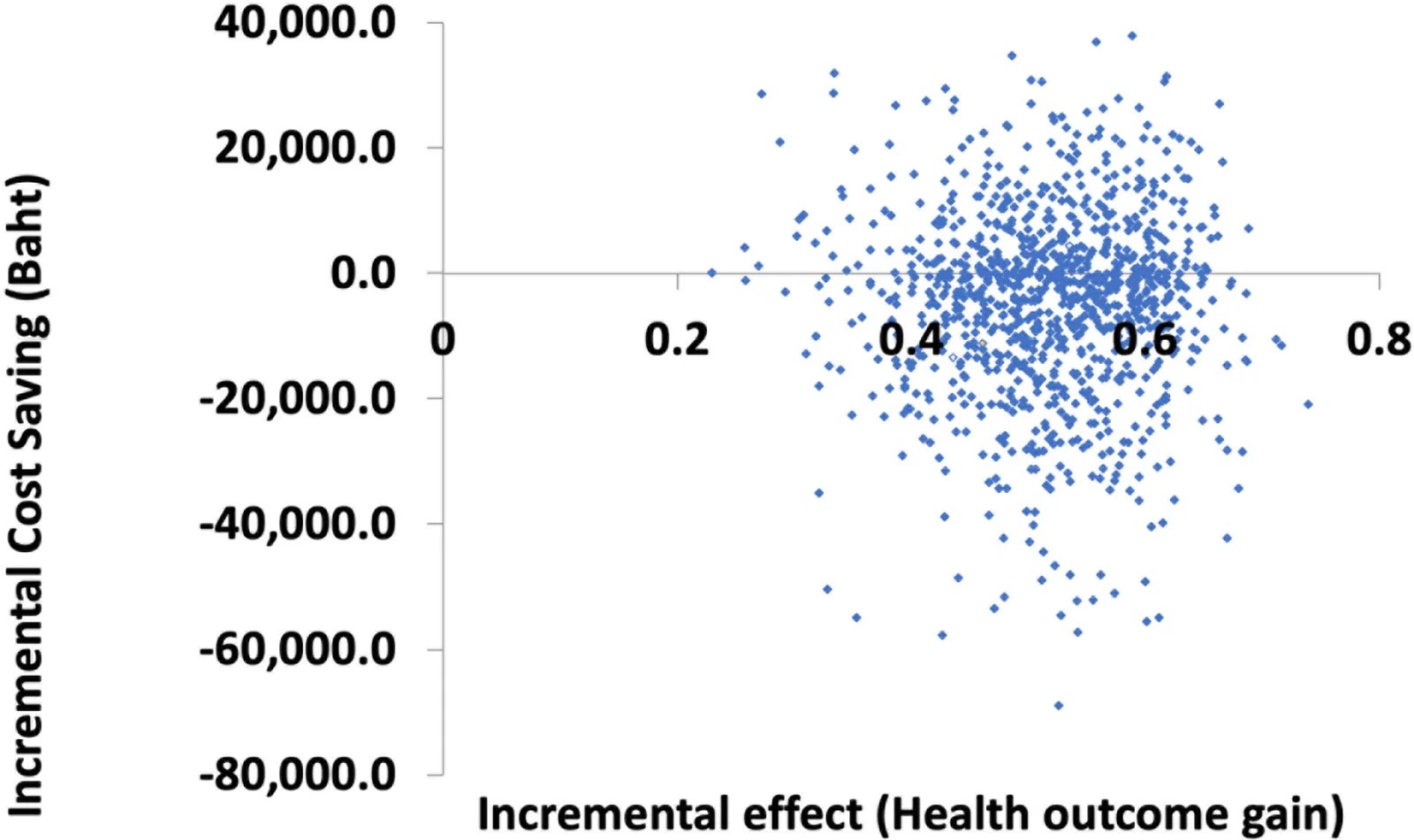
Simulation results on cost-effectiveness plane: providers’ perspective. Scatter plot of 1000 iterations simulation results on cost-effectiveness plane: providers’ perspective. Y-axis indicates incremental cost in Thai Baht. X-axis indicates incremental effect.

**Figure 4.**
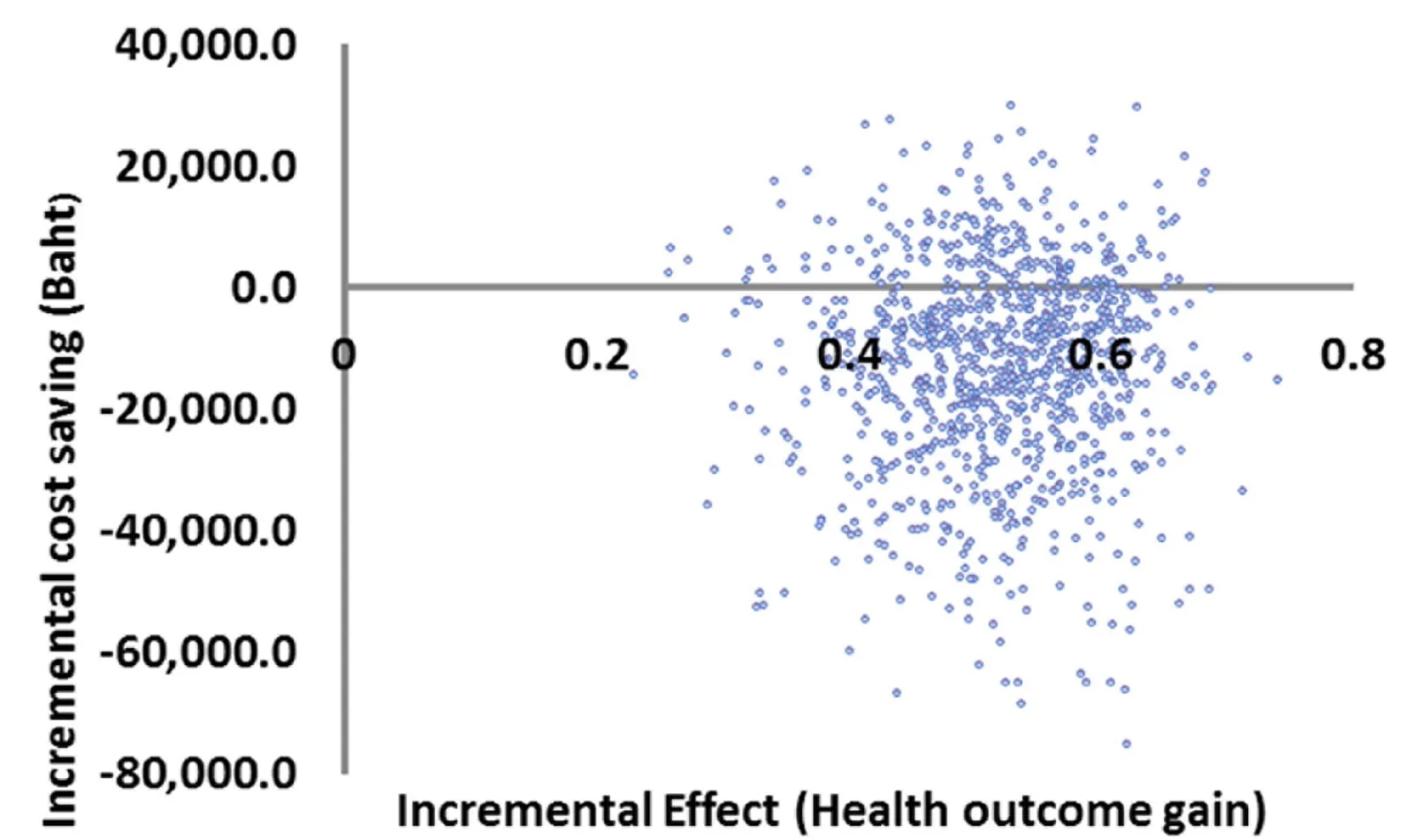
Simulation results on cost-effectiveness plane: patients’ perspective. Scatter plot of 1000 iterations simulation results on cost-effectiveness plane: patients’ perspective. Y-axis indicates incremental cost in Thai Baht. X-axis indicates incremental effect.

To further evaluate the robustness of the findings, a probabilistic sensitivity analysis was performed. As shown in Figures 3 and 4, 68.3% and 78.8% of the 1000 Monte Carlo simulations favored omeprazole plus standard treatment from the provider and patient perspectives, respectively.

## 4. Discussion

The ingestion of corrosive substances can have direct and indirect impacts on public health systems, as well as economic and social conditions.^[16]^ This study is the first to evaluate the cost-effectiveness of a standard treatment plus omeprazole compared to standard treatment alone for corrosive esophageal injuries Zargar grade 2b and 3a. The base case analysis indicated that omeprazole plus standard treatment was a dominant strategy, with lower costs and a lower observed stricture rate than standard care. This finding remained robust across the conventional one-way sensitivity analyses. However, the probabilistic sensitivity analysis favored the intervention in 68.3% and 78.8% of simulations from the provider and patient perspectives, respectively, indicating residual uncertainty regarding its economic advantage. Because omeprazole plus standard treatment was dominant in the base case analysis, with lower costs and a lower observed stricture rate than standard care, interpretation of the base case economic findings does not depend on a specific willingness-to-pay threshold. Therefore, a cost-effectiveness acceptability curve was not considered essential for the present analysis. From the provider perspective, omeprazole plus standard treatment resulted in cost savings of 4642.30 Baht per patient. From the patient perspective, the intervention resulted in cost savings of 5476.60 Baht per patient. Although the costs associated with this intervention may seem not so high, it can have a significant impact on individuals with low incomes. Additionally, if esophageal stenosis occurs, treatment costs can increase, along with other indirect expenses, loss of income, and reduced quality of life for both patients and their families. Some of these impacts cannot be quantified in monetary terms.

The study has some limitations that need to be discussed. Firstly, it was conducted in a single hospital, which is a large tertiary, government teaching hospital. Therefore, the costs obtained from this institution may not be representative of private hospitals. To address this concern, the researchers used deterministic and probabilistic sensitivity analyses to display the spread of expected values in comparison to private hospitals. Secondly, the study did not consider indirect costs such as loss of productivity and transportation costs. The transportation cost may significantly change the total cost of treatment, especially in patients whom refer from distant primary hospital. Moreover, the opportunity cost of time off work leads to a lack of income and an impact on families. Long-term follow-up costs and the lifetime impact of therapy for esophageal cancer risk after caustic injuries were not incorporated in the analysis. The study covered the immediate medical costs related to the intervention. The economic evaluation included only short-term medical costs during the study follow-up period. To further enhance the research, it is recommended to conduct a longitudinal study for more comprehensive details, including Quality-Adjusted Life Years. Furthermore, this economic evaluation was performed as a post hoc analysis of a previously completed RCT. As the economic analysis was not prespecified in the original trial protocol, the findings should be interpreted with appropriate caution because the economic evaluation was planned after completion of the clinical trial and was not prospectively designed to assess cost-effectiveness. Finally, the small sample size and short follow-up period limited our ability to adequately evaluate the long-term safety profile of omeprazole. Future studies with larger sample sizes and longer follow-up are warranted to further assess the long-term adverse effects of prolonged omeprazole therapy. In addition, although the intervention demonstrated a lower stricture rate, the small sample size resulted in wide confidence intervals around the treatment effect estimate. Consequently, the cost-effectiveness findings inherit the uncertainty associated with the underlying clinical effect and should therefore be interpreted with appropriate caution until confirmed in larger multicenter RCTs.

Patients and members of the public were not involved in the design, conduct, reporting, or dissemination of this economic evaluation.

This study suggests that omeprazole plus standard treatment may be a cost-effective strategy for adult patients with Zargar grade 2b and 3a corrosive esophageal injuries. However, larger multicenter RCTs are required to confirm these findings before widespread implementation.

## Acknowledgments

The authors sincerely thank Thammasat University Hospital for providing data on major service items and direct medical costs. We also thank Piyalamporn Havanond for the critical review of the study methodology and statistical analysis. Piyalamporn Havanond has provided permission to be acknowledged in this manuscript.

## Author contributions

**Conceptualization:** Prasit Mahawongkajit, Chittinad Havanond.

**Data curation:** Prasit Mahawongkajit, Saritphat Orrapin.

**Formal analysis:** Prasit Mahawongkajit, Saritphat Orrapin, Chittinad Havanond.

**Funding acquisition:** Prasit Mahawongkajit.

**Investigation:** Prasit Mahawongkajit.

**Methodology:** Prasit Mahawongkajit, Chittinad Havanond.

**Project administration:** Prasit Mahawongkajit, Chittinad Havanond.

**Resources:** Prasit Mahawongkajit.

**Software:** Prasit Mahawongkajit, Saritphat Orrapin.

**Supervision:** Prasit Mahawongkajit, Chittinad Havanond.

**Validation:** Prasit Mahawongkajit, Chittinad Havanond.

**Visualization:** Prasit Mahawongkajit, Prakitpunthu Tomtitchong, Chittinad Havanond.

**Writing – original draft:** Prasit Mahawongkajit.

**Writing – review & editing:** Prasit Mahawongkajit, Saritphat Orrapin, Prakitpunthu Tomtitchong, Chittinad Havanond.
